# Towards Autonomous Retail Stocking and Picking: Methods Enabling Robust Vacuum-Based Robotic Manipulation in Densely Packed Environments

**DOI:** 10.3390/s24206687

**Published:** 2024-10-17

**Authors:** Peter Kmecl, Marko Munih, Janez Podobnik

**Affiliations:** Faculty of Electrical Engineering, University of Ljubljana, Tržaška Cesta 25, 1000 Ljubljana, Slovenia; marko.munih@fe.uni-lj.si

**Keywords:** service robotics, retail automation, grasping, dense object detection, vacuum actuators, knowledge representation

## Abstract

With the advent of robotics and artificial intelligence, the potential for automating tasks within human-centric environments has increased significantly. This is particularly relevant in the retail sector where the demand for efficient operations and the shortage of labor drive the need for rapid advancements in robot-based technologies. Densely packed retail shelves pose unique challenges for robotic manipulation and detection due to limited space and diverse object shapes. Vacuum-based grasping technologies offer a promising solution but face challenges with object shape adaptability. The study proposes a framework for robotic grasping in retail environments, an adaptive vacuum-based grasping solution, and a new evaluation metric—termed grasp shear force resilience—for measuring the effectiveness and stability of the grasp during manipulation. The metric provides insights into how retail objects behave under different manipulation scenarios, allowing for better assessment and optimization of robotic grasping performance. The study’s findings demonstrate the adaptive suction cups’ ability to successfully handle a wide range of object shapes and sizes, which, in some cases, overcome commercially available solutions, particularly in adaptability. Additionally, the grasp shear force resilience metric highlights the effects of the manipulation process, such as in shear force and shake, on the manipulated object. This offers insights into its interaction with different vacuum cup grasping solutions in retail picking and restocking scenarios.

## 1. Introduction

Recent advancements in robotics and AI, such as the development of collaborative robots for seamless human–robot interaction [1], convolutional neural networks for dense object detection [2,3,4,5], and large language models to facilitate natural human–robot communication [6], have enabled task automation in human-adapted environments. Retail automation is a key area driven by growing demand and worker shortages, necessitating rapid development of robot-based technologies for repetitive tasks. Retail environments require robots to adapt to various scenarios like restocking shelves, customer assistance, and inventory management. Advanced robotic systems enhance efficiency, address labor shortages, and reduce operational costs [7]. Shelf stocking and item picking, which account for up to 60% of a store’s operational costs [8], are prime targets for automation. This can significantly improve speed, accuracy, and productivity in retail while creating more resilient supply chains and enhancing customer service through efficient order fulfillment.

Densely packed retail shelves pose unique challenges for robotic manipulation due to limited space between objects and diverse object shapes. Conventional finger-based systems often struggle in these environments due to the limited space between objects. Vacuum-based grasping solutions, unaffected by space constraints, offer an alternative but face challenges with varying object shapes and sizes.

Research in robotic grasping addresses the challenge of handling unknown objects. Miller et al. [9] proposed simplifying the process by breaking objects into basic shapes, reducing geometric complexity, and aiding grasp point determination. Saxena et al. [10,11] focused on enabling robots to handle unseen items and emphasized adaptability. The addition of a depth sensor [10] improved grasp stability by providing 3D shape information. Later, Saxena et al. [12] developed a system to estimate grasp positions for new objects and used images and depth sensors to perceive obstacles, plan, and execute grasps. Recent research in robotic grasping focuses on using neural networks to estimate grasps from 3D point clouds, thus bypassing the need to estimate object poses. Ten Pas et al. [13] introduced a successful method for grasping objects in cluttered scenes, though it required some human input or preprogrammed heuristics. To overcome this, *PointNetGPD* [14] was developed as an end-to-end neural network that generates grasps directly from point cloud data. The current state-of-the-art method [15] improves success rates and collision avoidance, making it effective in densely packed or random environments. Additionally, *Dex-Net 3.0* [16] advances suction cup grasping by computing robust vacuum suction targets from point clouds and using deep learning to identify optimal suction points for a variety of objects.

While grasping unknown objects has been well-studied, grasping in dense, ordered environments like retail shelves has seen fewer advancements. These settings, designed for humans, require robots to adapt to existing conditions. Thus, grasp planning algorithms must account for these constraints. The Amazon Picking Challenge tested these methods, revealing practical difficulties in robotic picking and areas needing improvement [17]. Building on this, Bormann et al. [18] developed a mobile manipulator for automated order picking, identifying detection and grasping challenges needing resolution for commercial use. Huang [19] tackled retrieving occluded objects from crowded retail shelves, using advanced search and manipulation strategies to enhance robotic performance in dense environments.

Vacuum grasping solutions address many retail environment challenges, but adaptability remains a key issue. One solution is combining vacuum with additional gripper deformations for enhanced versatility. While less researched than pressure-actuated systems common in soft robotics, vacuum actuators have shown promise. Studies on vacuum-actuated bending [20] and vacuum-powered soft mesh grippers [21] demonstrate their effectiveness in adaptive grasping. These actuators are versatile and efficient for handling diverse objects, making them suitable for retail environments. However, they share limitations with finger-based systems, such as difficulty in securely grasping certain objects and accessing items on densely packed shelves.

Advancements in dense object detection and knowledge frameworks are key to improving robotic systems in retail. Dense object detection allows robots to accurately identify and locate items on crowded shelves, thus enabling precise picking and stocking. Recent research in dense object detection has made significant advances. “Focal Loss for Dense Object Detection” [3] improves detection accuracy in dense environments by focusing on hard-to-detect objects. This is crucial for retail shelves where detectors often miss smaller or less-represented items. Goldman et al. [4] address this issue by lowering the detection score threshold to generate multiple detections and then using an EM (Expectation-Maximization) merging unit to combine them into single objects. This method enhances detection precision. They also provide the SKU-110K dataset, which contains dense retail shelf scenes, aiding in training and evaluating object detection methods for retail automation. The development of shelf-scanning robots and retail shelf datasets has made retail object detection increasingly relevant. Tonioni et al. [2] introduced an end-to-end solution that combines deep learning with classical object classification to improve product recognition accuracy and speed, which benefits automated inventory management. Fuchs et al. [22] demonstrated the effectiveness of CNNs for recognizing packaged products, highlighting their potential for retail product detection and classification. Guimarães et al. [23] reviewed the benefits of these methods for inventory management, order picking, and assisting visually impaired customers, showing the broader impact of advanced object detection in retail.

Ontologies provide a structured framework for categorizing and understanding a wide range of products, enhancing the robot’s decision-making and adaptability to various tasks. Ontologies enhance robotic systems by providing a knowledge-based framework for various tasks. Gayathri et al. [24] highlight the use of ontologies in domain modeling and path planning, which is essential for robots navigating complex environments. Paulius et al. [25] underscore the importance of standardized knowledge representation models in service robotics, emphasizing the construction of comprehensive models from smaller, modular fragments to enhance robots’ autonomy and efficiency in various tasks.

Recent studies have explored various approaches to robotic manipulation in retail environments. Spahn et al. present a mobile manipulator system capable of adaptive object picking and placement in human-shared spaces, focusing on task planning, interaction, teaching, and safety [1]. Although their system demonstrates an end-to-end solution for object manipulation, they emphasize the need for further advancements, particularly in gripper design and mobile manipulator technology. Similarly, Garcia Ricardez et al. introduce a robotic system designed for restocking and straightening items on retail shelves, utilizing a new gripper design with a suction cup and additional degrees of freedom [26]. They highlight the importance of precise item placement and shelf appeal as these factors significantly influence customer purchase decisions. These studies point out the challenges and potential improvements in retail automation, aligning closely with the objectives of our proposed system.

In this paper, we present a robotic system for object manipulation on retail shelves. This system integrates a LIDAR, an RGB camera, a knowledge representation model, and adaptive vacuum suction cups, building on concepts similar to those in Tonioni et al. [2]. Next, we aim to demonstrate the success rate of our system through comprehensive testing and evaluation. Lastly, our goal is to provide a benchmark for similar systems in the future, offering a standard against which new developments can be measured.

The paper is structured as follows: Section 2 details our system and experimental setup, explaining the integration and functionality of each component. Section 3 presents the data we have gathered during our experiments, providing a thorough analysis of our system’s performance. Section 4 discusses the implications of the data, highlighting key findings and areas for improvement. Finally, Section 5 offers concluding remarks, summarizing the achievements of our research and suggesting directions for future work.

## 2. Materials and Methods

In this section, we discuss the proposed system architecture and experimental setup, including the used equipment. We pay special attention to our novel adaptive vacuum suction cups, our vision and grasp selection pipeline, experimental procedures, and evaluation metrics. We also propose a new metric for measuring sheer force resilience in lateral access vacuum-based grasping scenarios such as retail shelf picking and restocking.

### 2.1. Proposed System Architecture

A retail store’s complex environment presents many challenges for robotic applications due to the variety of objects, human-centric layouts, and densely packed objects on the shelves. This requires a sophisticated robotic system with several sub-modules. While our primary goal was to develop a minimal working system (see Section 2.2), here we propose a comprehensive robotic system for several reasons.

First, the system demonstrates all the core requirements for a fully autonomous store assistant, setting the foundation for further research and development. The modular architecture ensures that the current system can be extended to meet the long-term goal of full store automation.

Second, this modular framework enables us to develop and refine specific sub-modules independently, allowing for targeted improvements in tasks and sub-tasks such as object detection, reasoning, grasping, and navigation. This flexibility also allows us to swap modules to address different experimental scenarios, for instance, replacing the object detection sub-module when adapting the system to different store types, such as clothing, electronics, or home improvement stores, without having to re-implement the object validation system.

Third, this approach enables us to focus our efforts on improving specific modules where further development is needed while relying on established solutions in areas where research is already extensive.

**Figure 1:**
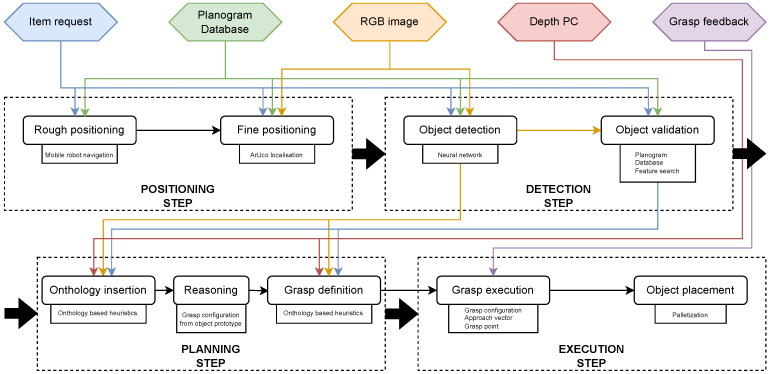
Proposed system architecture, data flow, and program flow. Each discrete step in the program flow is marked with a dashed outline; their sequence is marked with thick black arrows. The data flow and internal sequences are marked with thin arrows. Black arrows represent general program flow with uncategorized data exchange. Colored arrows represent program and data flow where specific input or output data are present.

The system includes a mobile robot base, a manipulator with a suitable grasping solution, a computer with wireless connectivity for offline image processing, and various sensors for object detection and grasp execution. The proposed tasks include positioning, detection, planning, and execution, which are further divided into sub-tasks. The full system architecture is shown in Figure 1.

The system operates in four main steps:

1. *POSITIONING STEP* (Figure 1): The robot system receives an item request from the store management system. It queries a planogram database for the item’s location, and the robot’s mobile base navigates to the shelf. As the planogram database was not designed for use with robotic systems, the rough position achieved after this step might not be sufficient for object detection or manipulation. For precise positioning, ArUco markers on shelf price sections are used, with different markers for closely spaced shelves to prevent errors.

2. *DETECTION STEP*: The robot detects objects on the shelf, segments the requested item, and compiles an object description. The object description contains all the data from the planogram database (size, weight, material), as well as additional information acquired during the second step (RGB, depth PC, and their derivatives). Using its manipulator, it captures RGB images and depth point clouds of the shelf area according to the planogram. Objects are segmented and validated against planogram data using heuristics and feature matching, similar to Tonioni et al. [2]. If the correct object is found, the system retrieves additional parameters from the planogram database, such as exact size, packing material, and weight. This information, along with the cropped depth PC and RGB image, is passed on to the planning step via the object description. Details on the neural network, feature search and object description are described in Section 2.4.

3. *PLANNING STEP*: The system selects an object prototype based on heuristic parameters from the complex description and ontology model. It then reasons about the best grasp type for the object and scenario. The system further parameterizes the grasp using heuristic information from the grasp instance. Further explanation of the onthology and grasp selection is presented in Section 2.4.

4. *EXECUTION STEP*: The system validates grasp reachability, generates approach vectors and positions, and executes the grasp while monitoring sensor feedback (force, air pressure, distance, speed). After a successful grasp, the object is placed on the mobile platform using a palletising algorithm. For restocking, the robot identifies empty shelf spaces, detects and grasps objects from its base, and places them on the shelf.

### 2.2. Experimental Setup

**Figure 2:**
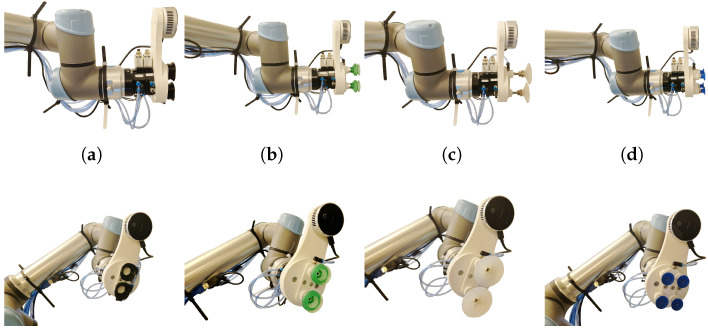
Layout and type of suction cups used in experiments. Suction cups (**a**) are our novel suction cups. Suction cups (**b**) feature an accordion structure to enable grasping of more object types. Suction cups (**c**) feature the biggest diameter. Suction cups (**d**) have the smallest diameter, which allows us to place them in a matrix.

Our experimental setup consists of a mobile robot platform, a UR5 robot manipulator (Universal robots, Odense, Denmark), an Intel L515 LIDAR and RGB camera (Intel, Santa Clara, CA, USA), and a grasping tool using suction cups, discussed further in Section 2.3. Commercially available suction cups and novel adaptive vacuum suction cups were used. The types and layouts of the suction cups can be seen in Figure 2. During experimentation, various retail items and reference objects are positioned in a dense distribution on standard retail shelving, as seen in Section 2.5. Additionally, accelerometers are attached to the retail objects and robot flange to enable the measurement of sheer force impact on the manipulated retail object.

### 2.3. Vacuum-Based Grasping Tool

**Figure 3:**
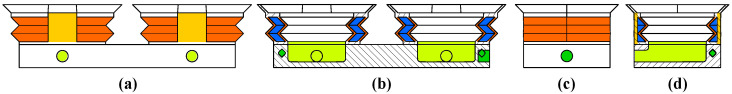
Sketch of our proposed adaptive vacuum grasping tool. Images (**a**,**c**) show the external structure. Images (**b**,**d**) depict the internal structure, including the vacuum channels in the base (green), the deformation chamber (blue), the deformable (orange) and non-deformable (yellow) walls, and the primary vacuum inlet (lime).

The experimental setup includes our vacuum-based grasping tool. The tool is based on a suction cup design, which is able to adapt its shape to better accommodate curved objects. The shape adaptation is possible with the help of a chamber that deforms specific walls of the suction cup, thus introducing a curvature to the seal when the vacuum is applied. The chamber is emptied via an additional inlet on the side of the suction cup’s base and a channel running through it. To achieve the complex shape of the suction cup and its base, we utilized 3D printing technologies (FDM) for fabrication. A sketch with highlighted functional parts is depicted in Figure 3.

**Figure 4:**
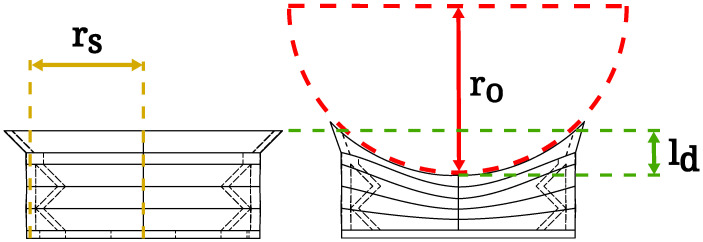
The suction cup, presented in its initial state (**left**) and with applied vacuum to the vacuum chamber–deformed state (**right**). The red semicircle depicts a cylindrical object with radius $r_{o}$. The green lines represent a linear deformation of the suction cup’s accordion walls $l_{d}$. The yellow lines show the radius of the deformable part of the suction cup $r_{s}$.

The curvature of the suction cup’s seal is dependent on the linear deformation of its accordion sides (Figure 3, orange part), caused by the vacuum supplied to the vacuum chamber (Figure 3, blue part). The sides with the accordion structure deform with supplied vacuum as defined in Equation (1),

(1) $$l_{d}=k_{d}P$$

where $l_{d}$ is defined as the linear deformation of the suction cup’s side in millimeters, $k_{d}$ is the factor of deformation in millimeters per pascal and *P* is the supplied vacuum in pascals. In the case of our suction cup geometry and material (TPU), the factor of deformation is equal to 0.074 mm/kPa. While the accordion sides deform, the rigid sides remain in their initial state, introducing a curvature to the top part of the suction cup and the seal. The curvature of the seal can be defined in regard to the linear deformation with Equation (2),

(2) $$r_{o}=\frac{4l_{d}^{2}+r_{s}^{2}}{8l_{d}}$$

where $r_{o}$ is the radius of the object the suction cup’s seal can now adhere to, $l_{d}$ is the linear deformation of the suction cup’s accordion sides in millimeters, defined in Equation (1), and $r_{s}$ is the radius of the suction cup. The variables, presenting physical properties of the suction cup and object, used in Equations (1) and (2) are illustrated in Figure 4. These equations can be used during the grasp parameterization step to ensure the best possible contact of the suction cup’s seal to the manipulated object.

**Figure 5:**
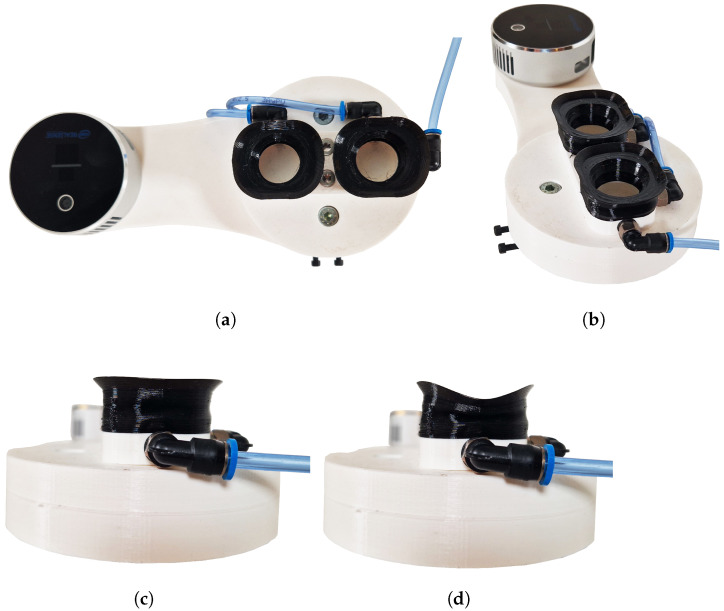
Top view (**a**), birdseye view (**b**), and side view (**c**,**d**) of our novel suction cups. Images (**a**–**c**) show the suction cups without any vacuum applied to the deformation chamber, while image (**d**) presents the fully deformed form.

For the purpose of the proposed experiments, we designed a modular tool to hold our novel suction cups, any commercial suction cups, as well as the camera and LIDAR combo unit. The suction cups were printed with the FDM technology in TPU (shore hardness 95A). An image of the tool from different perspectives, as well as the suction cups in the deformed and non-deformed state, can be seen in Figure 5.

### 2.4. Detection System and Grasp Planning

The detection system processes RGB-D images captured by an Intel L515 LIDAR camera to locate and identify store items. The pipeline combines neural network-based object detection, feature search validation, point cloud processing, ontology-based reasoning for grasp selection, and geometry-based grasp parametrization.

The detection pipeline starts by capturing RGB-D images using the Intel L515 LIDAR camera. The RGB image is processed by a neural network based on the VFNet architecture [5], implemented using the mmdetection 2.12.0 Python package with PyTorch 1.5.0 and Python 3.7.17. The network is trained on the SKU110K dataset [4], which includes over 1.7 million annotated bounding boxes from various retail environments, ensuring robustness against different scales, angles, lighting, and noise levels. The trained neural network was validated using images captured from local supermarkets. The network generates initial bounding boxes that indicate the locations of potential store items, but these do not yet specify the items’ identities. The bounding boxes are then refined using an Expectation-Maximization (EM) algorithm described in Goldnam et al. [4] to improve detection accuracy by adjusting box parameters.

The system then uses the robot’s position and information from the store’s planogram database to hypothesize the most likely items on the shelf. The planogram provides details like packaging, size, weight, and reference images. A feature search algorithm validates these hypotheses and selects the requested item by using MSER feature search and SIFT descriptors to compare objects inside the bounding boxes and reference images. This combination was proven to be effective in various lighting conditions and with various affine transformations between the reference and captured images [27].

After validation, the relevant section of the point cloud is extracted and filtered for outliers. An object description is then compiled, incorporating the cropped RGB image, filtered point cloud, planogram reference images, and item details such as size, weight, and packaging material. The compiled object description is passed to an ontology reasoning system.

The ontology defines a structured representation of various object classes, instances, and grasp types, along with the relationships between them. It classifies both object and grasp instances to allow the selection of a suitable grasp for the identified item in the current scenario. The reasoning system employs symbolic logic; for example, an object with a parameter indicating a flat front will prompt the selection of a grasp suitable for flat surfaces.

After the grasp type selection, the final grasp is parameterized using point cloud data and packaging information. The parametrization includes the selection of a grasp point, an approach vector for the robot, as well as any additional parameters the grasp type requires (for example, the suction cup curvature, supported by our new suction cups). For example, when grasping objects with flat fronts, the most suitable grasp point was determined to be the object’s centerline, with a straight-on approach and a flat suction cup. For curved objects, the suction cup is adjusted based on the object’s radius, the grasp point is selected from fitting a curve onto the pointcloud, selecting the frontmost section (equivalent to the centerline), and the approach vector is generated from the tangential plane of the curvature.

By integrating these steps, the detection system enables the robot to accurately locate, identify, and grasp items in various conditions, optimizing the retrieval process.

### 2.5. Experimental Procedures

Due to the complexity of the proposed architecture, we decided to limit our experiments to steps 2 through 4, focusing primarily on object manipulation. Four experiments were conducted:

1. Section 2.7: Explores inaccuracies in vision and depth-based detection systems. Aim is to only analyze the performance of detection phase.
2. Section 2.8: Demonstrates effectiveness of various suction cups, showing robustness against detection errors.
3. Section 2.9: Examines success rates of grasping solutions on different object types.
4. Section 2.10: Compares grasping solutions for shear force resilience and grasp stability, introducing a new metric for grasp quality during high-acceleration movements.

All experiments consist of one or more of the following phases:

- **Grasp point definition:** During this phase, a grasp point and an approach vector are generated (system architecture step 3) with the help of our detection system (system architecture step 2). Depending on the used grasping solution, the grasp can be parameterized depending on the detected object. In some experiments, the grasp point and approach vector are predefined in order to eliminate the detection system’s influence.
- **Grasping:** During the grasping phase (system architecture step 4), the manipulator approaches the grasp point from the direction of the approach vector. Force and vacuum sensors are used to detect a successful grasp. Due to the variety of objects and grasping solutions, recovery behaviors are implemented to ensure predictable behavior in the case of an unsuccessful grasp.
- **Manipulation:** The manipulation phase (system architecture step 4) is designed to test the behavior of grasped objects during acceleration and deceleration. This phase mainly consists of simple linear and rotational movements followed by a placing operation.
- **Experiment reset procedure:** After each experiment iteration, a reset procedure is run if the experiment resulted in an object being moved from its initial position. The reset procedure may use a random variable to ensure variety in starting conditions.

In experiments where a grasp point is not defined as a constant (the second experiment), we use the LIDAR and camera combo to determine grasp points, approach vectors, and additional grasp parameters (see Section 2.3), as described in the proposed system architecture (Section 2.1, step 2 (detection) and step 3 (reasoning)). The RGB data are passed to a neural network based on the VarifocalNet [5] backbone with an EM merging unit [4] as a post processor. The result is a bounding box, which is used to crop the depth information to include only object depth data and exclude its surroundings. A simple heuristic method differentiates between round and square objects, and a plane or cylinder fitting algorithm retrieves a possible grasp point as well as object parameters (height, width, radius, etc.). The parameters are fed into an ontology, linking the parameterized object to a parameterized grasp definition, enabling the use of complex grasping solutions.

During the experiments, we gathered data from the robot using the RTDE (Real Time Data Exchange) protocol, including robot Cartesian position, velocity and acceleration, force and torque on the robot’s TCP, and the current step of program execution. These data were gathered at a rate of 500 Hz. Data from the 3D accelerometer were gathered at a rate of 100 Hz. Whenever the camera is required to capture an image, the RGB and depth data are also gathered, alongside the detected object positions and point clouds.

**Figure 6:**
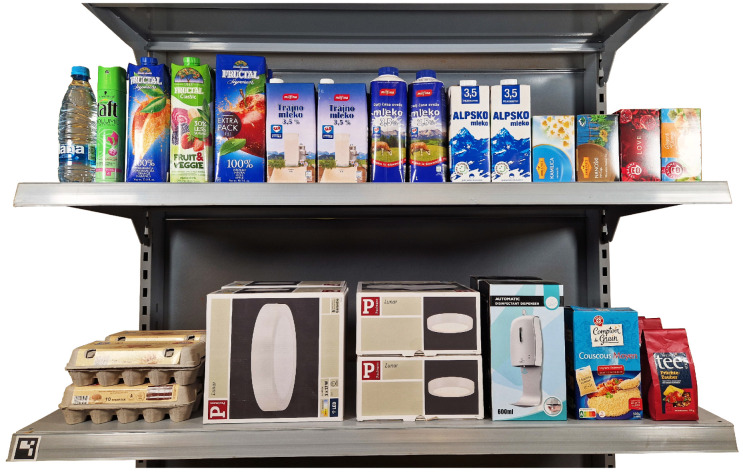
The experimental setup of retail shelves. Objects are positioned in a dense configuration to emulate real retail store conditions.

**Figure 7:**
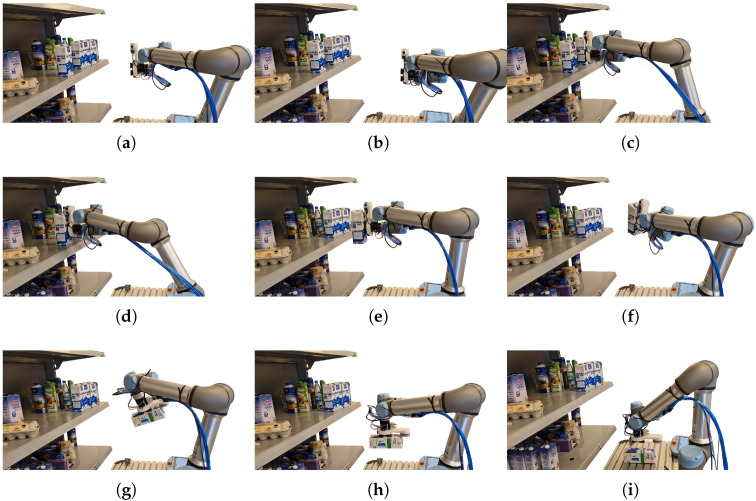
The sequence of experimental procedures. Image (**a**) depicts the robot in its home position before manipulation. On Image (**b**), the robot moves to the image capture position and processes the image. Image (**c**) shows the robot in the approach position before the grasp point. Image (**d**) displays the grasp. The robot then moves to the position in Image (**e**) using a linear movement. After the linear movement, a combination of rotational movements is performed, as seen in images (**f**,**g**). Lastly, another linear movement, seen in Image (**h**), is used to place the object in its final position, as seen in Image (**i**).

The experiments are conducted using various retail items placed on standard retail shelving to simulate real-world conditions, as seen in Figure 6. We utilize four different types of suction cups to test the grasping tool’s performance across different scenarios. The suction cups and their configurations can be seen in Figure 2. In the case of our suction cups, we change the curvature of the suction cups to fit the object. The trajectories executed by the robot after grasping are consistent across all trials, ensuring that the results are comparable. The trajectories are split into multiple phases, described in Figure 7. The phases included in our measuring results are a linear movement along the X-axis (Figure 7e), a mixed rotational movement along all axes (Figure 7f,g) and a linear movement along Z-axis (Figure 7h).

### 2.6. Reference Objects

For our experiments, we used a combination of real retail store items and reference objects designed to ensure repeatable conditions for grasping. The reference objects were 3D printed in PLA and smoothed.

**Figure 8:**
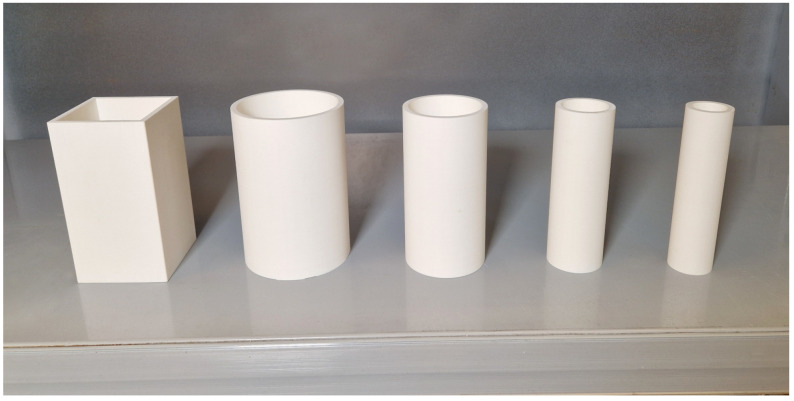
The reference objects of different shapes and sizes. From left to right: flat object with 80 mm side, curved object with radius 100 mm, curved object with radius 75 mm, curved object with radius 50 mm, and curved object with radius 37.5 mm. All objects have a height of 150 mm.

We chose an array of different shapes and dimensions that would enable us to emulate as many retail objects as possible without requiring too many different reference objects. Focusing mainly on objects with well-defined shapes, we chose one-sized square objects with a side of 80 mm and four sizes of cylindrical objects with diameters of 37.5 mm, 50 mm, 75 mm, and 100 mm. All objects have the same height at 150 mm. The objects are hollow and have an opening at the top. This enables us to test different object weights as well as weight distributions and their effects on the grasp performance. All reference objects can be seen in Figure 8.

### 2.7. Detection Error

Before evaluating our grasping solution, we required a baseline measurement of detection capabilities. Alongside the success rate of object detection, we identified object position error as a key performance metric for analyzing different types of suction cups compatibility.

The experiment was conducted with various convenience store items (refer to Figure 6). The items were placed on a same fixed position on the shelf, and detection tasks were performed from various camera positions. The results were processed with the algorithm mentioned in Section 2.4 and Section 2.2, to determine the object’s most suitable grasp point (center of the object) and approach vector. The grasp points were transformed to the robot’s global coordinate system and used to visualize the measurements’ spread. The maximum deviation from the average was used to assess the ability of the grasping solutions to adapt to detection errors (see Section 4).

### 2.8. Grasping Robustness Against Detection Error

To evaluate the grasping solution’s robustness against detection error of the position of the object, we designed an experiment to identify the maximum deviation from the most suitable grasp position at which a grasp is still successful. By using reference objects, we eliminated the variability introduced by different material properties, ensuring that any observed effects were due to the grasping solution’s performance.

The objects were placed in a known position on standard retail shelving. The robot then executed a grasping action on the known position, with random linear or rotational offsets applied along the X and Y axes. The Z-axis offsets, both linear and rotational, were deemed unimportant since the objects remained upright, and the vision system, aided by the LIDAR, provided accurate distance measurements from the robot’s TCP (tool center point). The linear offsets ranged from −50 mm to 50 mm, and the rotational offsets ranged from −5° to 5°. The grasp was counted as successful if the robot grasped the object and placed it in a designated area. The results of this experiment include the maximum deviations for each axis that still enabled a successful grasp of either flat or curved objects for different grasping solutions.

### 2.9. Grasping Success Rate

To assess the grasping success rate for each suction cup, we performed successive grasps of real retail store items. A grasp is counted as successful if the robot can grasp the item and move it to a designated position. The success rate is presented for different cup types and grasped objects (either flat or curved). If a grasping cup and item type combination did produce a success rate of 0%, the experiments would be marked as not applicable as the grasping cup is not capable of grasping this type of item. An example of an experimental procedure used to test the success rate can be seen in Figure 7.

### 2.10. Grasp Shear Force Resilience

Assessing the impact of shear forces on object stability during manipulation led us to propose a new metric called Grasp Shear Force Resilience. This metric is derived from the difference between the acceleration norms measured on the object and the TCP of the robot with a 3D accelerometer. The difference is squared to ensure a positive value, and a mean over N samples is taken. We take the inverse root of the result to revert to the linear scale and make sure that a bigger value represents a bigger shear force resilience. This method is further presented with Equation (3):

(3) $$gsfr=\frac{1}{\sqrt{\frac{\sum_{i=1}^{N}\left(|\right|a_{obj,i}\left|\right|-\left|\right|a_{flange,i}{\left||\right)}^{2})}{N}}}$$

where $\left|\right|a_{obj,i}\left|\right|$ is the i-th acceleration norm of the accelerometer attached to the object, $\left|\right|a_{flange,i}\left|\right|$ is the i-th acceleration norm of the accelerometer attached to the robot flange, *N* is the number of samples and gsfr is the grasp shear force resilience factor. In our experiments, the acceleration data are from the collected accelerometers attached to the objects and robot flange.

## 3. Results

This section includes the results of each experiment. Firstly, we present the data gathered from the detection system, analyzing its error. Next, we include the data gathered from the grasping reference objects and comment on the robustness of error detection for each suction cup. Lastly, we will present the data gathered during grasp shear force resilience testing and their corresponding metrics.

### 3.1. Detection Error

**Figure 9:**
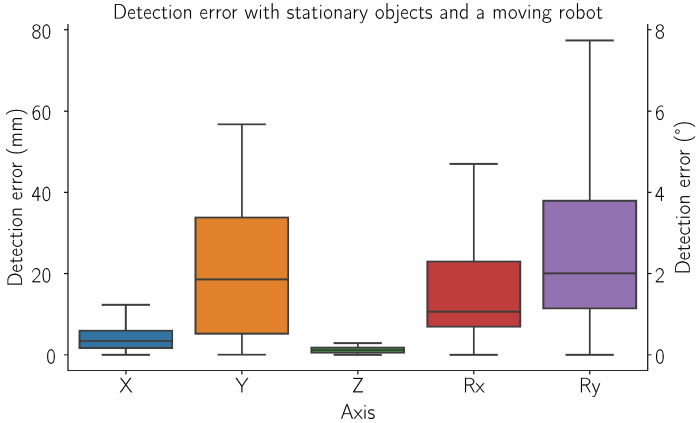
Detection error with moving robot. The robot moved in a rectangular pattern with 7 × 5 × 3 points where it stopped and captured image data. The error is presented for different axes (in mm for translational and in ° for rotational).

Figure 9 shows the error of the detected object position and orientation for different real retail store objects. Figure 9 shows the boxplot for detection error if the stationary object is observed from different points around the shelf. Data are presented in five columns, each representing an axis of detection. For columns denoting a linear axis, millimeters (mm) are used, while rotational axes use degrees (°). In this case, the robot moves over a grid of points (7 × 5 × 3, for a total of 105 points). The experiment was repeated 15 times for each grid point.

### 3.2. Grasping Robustness against Detection Error

In this section, we present the robustness of different suction cups against detection errors generated by the detection method. We present the maximum error from the most suitable grasp point, where the grasp of a reference object is still successful.

**Figure 10:**
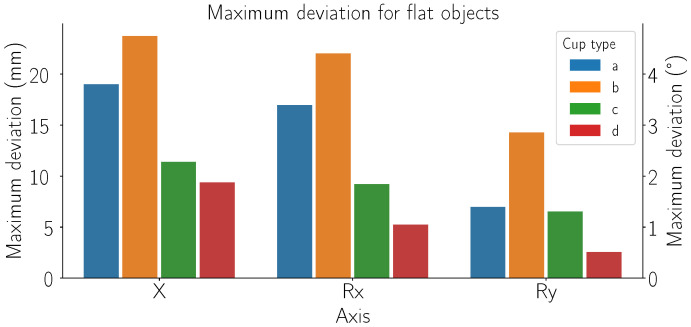
Maximum deviation from the most suitable grasp point, where the grasp of a reference object is successful, for each grasping cup. Plot groups represent tested axes, each color depicts the cup type (refer to Figure 2 for list of grasping cups). The units used are mm for translational axes and ° for rotational axes.

In Figure 10, we present the maximum deviation measured while grasping flat objects (specifically the flat 80 × 80 mm reference object). Each suction cup is marked with its own color and letter refer to Figure 2 for a list of grasping solutions). Each group of graphs denotes a different axis where the error was applied. The graphs present the maximum deviation from the most suitable grasp point in millimeters (mm) for linear axes and degrees (°) for rotational axes.

**Figure 11:**
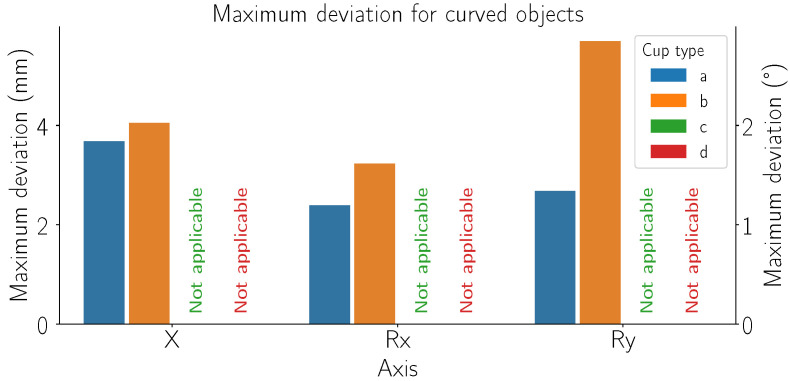
Maximum deviation from the most suitable grasp point for curved objects. Plot groups represent tested axes, each color depicts the uncertainty compensation ability for a specific grasping solution (refer to Figure 2 for list of grasping solutions). The units used are mm for translational axes and ° for rotational axes.

Figure 11 shows the maximum deviation measured while grasping curved objects (specifically the curved 75 mm reference object). Due to the shape of the suction cups (c) and (d), we were unable to measure the maximum deviation as they were unable to successfully grasp the curved object.

### 3.3. Grasping Success Rate

**Figure 12:**
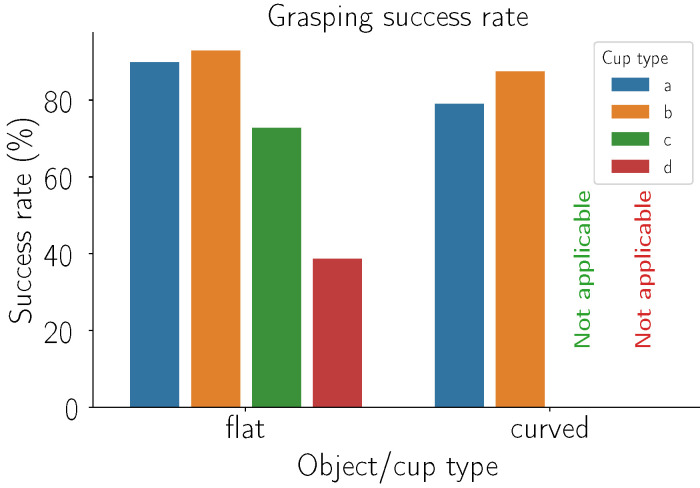
Success rate of different cups grasping different objects. Each group of bar graphs represents a type of object the robot was trying to grasp, while the different colors represent different cups used (refer to Figure 2 for a list of cups). Data for grasping curved objects with cups (c) and (d) are not available, as the mentioned grasping solutions are not suited for grasping them.

This section presents the grasping success rate of grasping real retail store objects with different cups while relying on our detection framework. Figure 12 presents the percentage of successful grasps for each cup either for flat or curved objects. For cups that could not grasp a curved object, the data are marked as “Not applicable”. For each object/cup pair, 60 repetitions were made.

### 3.4. Grasp Shear Force Resilience

In this section, we present results from our novel evaluation metric called grasp shear force resilience. The metric is derived from acceleration data as described in Section 2.10.

**Figure 13:**
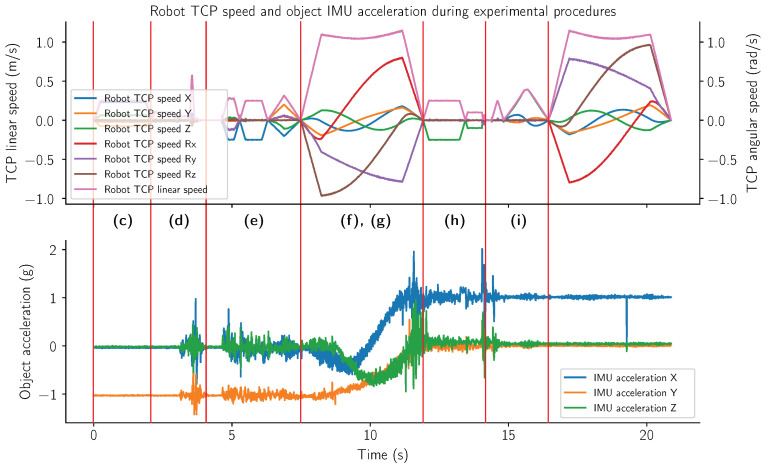
TCP axis speeds (**top**) and object accelerations (**bottom**) during experimental procedures. The phases of experimental procedures are separated by red vertical lines and marked with letters corresponding to Figure 7. The top graph is provided in m/s for linear movements and rad/s for rotational movements. The bottom graph describes the acceleration in gs, where 1 g is approx. 9.81 m/s^2^.

Figure 13 shows the data during different stages of the experimental procedure as described in Figure 7. The letters correspond to the aforementioned experimental phases, which are separated by red vertical lines. The top graph presents the robot velocities during the experimental procedure in either meters per second (m/s) for linear axes or radians per second (rad/s) for rotational axes. The bottom graph presents the acceleration data captured by the accelerometer attached to the object. The graph is presented in a multiple of average gravitational acceleration (g), measuring approximately 9.81 m/s^2^.

**Figure 14:**
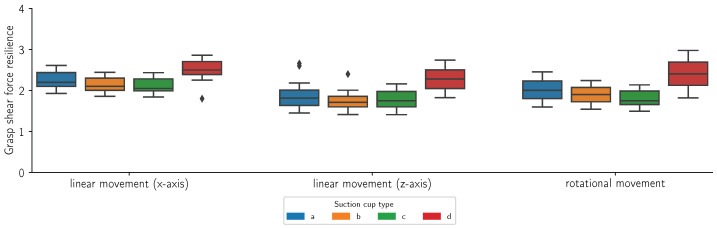
Grasp shear force resilience measurements (n = 60) for a square retail object with a mass of 1 kg during a linear motion along the x-axis, a linear motion along the z-axis and a mixed rotational movement. The colors refer to different suction cups (refer to Figure 2 for a list of suction cups).

**Figure 15:**
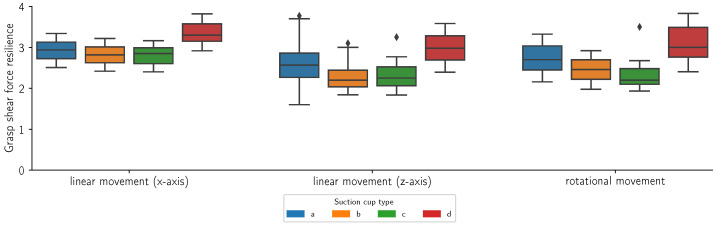
Grasp shear force resilience measurements (n = 60) for a square retail object with a mass of 0.5 kg during a linear motion along the x-axis, a linear motion along the z-axis and a mixed rotational movement. The colors refer to different suction cups (refer to Figure 2 for a list of suction cups).

**Figure 16:**
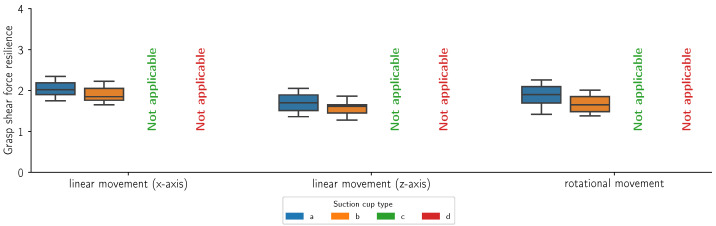
Grasp shear force resilience measurements (n = 60) for a cylindrical (curved) retail object with a mass of 1 kg during a linear motion along the x-axis, a linear motion along the z-axis and a mixed rotational movement. The colors refer to different suction cups (refer to Figure 2 for a list of suction cups).

**Figure 17:**
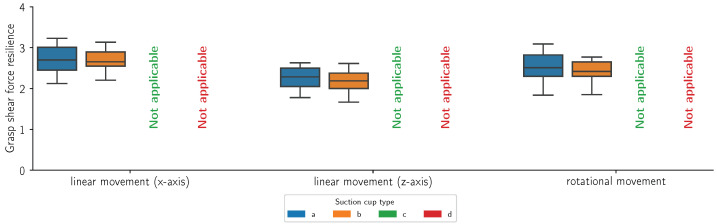
Grasp shear force resilience measurements (n = 60) for a cylindrical (curved) retail object with a mass of 0.5 kg during a linear motion along the x-axis, a linear motion along the z-axis, and a mixed rotational movement. The colors refer to different suction cups (refer to Figure 2 for a list of suction cups).

Figure 14, Figure 15, Figure 16 and Figure 17 present the grasp shear force resilience factor (higher is better) for different grasping solutions (refer to Figure 2 for list of types of suction cups), manipulating different flat and curved, retail and reference objects of weights 0.5 kg and 1 kg during linear motions along the X (first group) and Z axes (second group) and a combined rotational movement around all axes (third group). Each movement was repeated 60 times per object and cup combination. The colors denote different suction cups: orange, green, and red are commercial suction cups and blue is our novel suction cup. Data are presented in boxplot form. The metric provides comparative results and does not bear a unit. Where measurements were not possible due to limitations of the suction cup, the data are marked as “Not applicable”.

In order to properly determine the statistical relevance of measured data, we utilized the Mann–Whitney U test to retrieve the *p*-values corresponding to pairs of data retrieved from experiments with our novel grasping solution and the commercial grasping solutions. These data are presented in Table 1. Data pairs with *p*-value under 0.05 are deemed borderline statistically relevant and are presented in italic form, data pairs with *p*-value under 0.01 are deemed statistically relevant and are presented in bold form.

## 4. Discussion

The detection error experiments were conducted using the detection pipeline of our proposed framework. It is similar in design to a work by Tonioni et al. [2]; however, we implemented a different CNN for dense object detection—VFnet (Zhang et al. [5]) combined with an EM merger unit for postprocessing (Goldman et al. [4]), as well as additional features like heuristics for best object selection, LIDAR for capturing depth data, and a method to determine the best grasp point and approach vector for a robot.

Our grasping pipeline draws inspiration from several key works, including those by Miller et al. [9], Saxena et al. [10,11,12], and Mahler et al. [16]. Specifically, Miller et al. [9] introduced an approach that utilizes shape primitives to identify optimal grasp points based on object geometry, which bears similarities to our method. However, a significant distinction lies in our use of ontologies, which allows us to categorize objects based on shape, weight, material, and other properties, thus enabling the selection of grasps that are not solely shape-dependent. The work by Saxena et al. [10,11,12] presents a less direct comparison as it focuses on generating grasp targets in cluttered environments based on visual data. While this approach has potential relevance, it is primarily designed and evaluated for cluttered scenes, which, though structurally complex, are less dense. As noted in related research, techniques optimized for cluttered scenarios often face challenges in dense settings, which are more representative of our application. The most comparable work is that of Mahler et al. [16], which involves generating vacuum grasp targets from point clouds. Although this closely aligns with our goals, Mahler’s work primarily deals with grasping isolated objects of higher geometric complexity than our targets. Despite these differences in scenario and object complexity, performance comparisons between the two approaches can still be drawn.

Figure 9 shows the effect of robot position on the detection error, a visible difference from the other measurements is the Z-axis, which is particularly small. This is due to the detection error being primarily dependent on the LIDAR’s uncertainty, which is on the order of 5 mm. In contrast, the Y-axis uncertainty is almost four times larger than the X-axis. This difference arises from detections where part of the top and bottom of the object are not included in the detection bounding box. However, this is not particularly problematic, as the objects are usually upright, allowing for greater deviation of the center in the Y direction. The primary source of error in the X-direction is likely associated with changes in the camera’s perspective. This issue could be mitigated by performing two detections: one before and one after the identification of the object, with particular attention to maintaining consistent camera positions relative to the identified objects. Additional errors may arise from irregular object shapes or protrusions on the packaging, which can subtly alter the grasp plane. These issues can be addressed with improved filtering or point cloud segmentation, focusing on features critical for grasp generation. Furthermore, inconsistent lighting conditions may contribute to detection errors. Although our experiments were conducted under varying lighting due to the time of day or weather, we observed no significant differences in the captured data in most cases. Nonetheless, adding a dedicated light source to the manipulator could yield noticeable improvements in real-world applications, given the diverse range of objects and shelving conditions. It is important to note that real in-store environments typically have more consistent lighting.

The suction cup experiments were designed to evaluate our solution and compare it to commercially available suction cups, highlighting the unique adaptability of our design. Unlike the suction cups used by Spahn et al. [1], which are mounted on a long pole to improve lateral access in retail environments, our suction cups utilize a vacuum mechanism that allows them to change shape and conform to a variety of object geometries, enhancing their grip on both flat and curved surfaces. While Spahn et al. [1]’s focus was on extending reach rather than grip adaptability, Garcia Ricardez et al. [26] added degrees of freedom through a swiveling and extending base to improve manipulation in tight spaces. However, their design still prioritizes maneuverability over object adaptability.

As shown in Figure 10, our adaptive cups (a) allow large maximum deviation when handling flat reference objects (80 × 80 mm). Specifically, it can effectively manage deviations up to 18 mm in the X direction, 3 degrees of rotation around the X-axis, and 1 degree around the Y-axis. These results are significantly better compared to cups (c) and (d) and are slightly inferior to grasping solution (b). The best performance of solution (b) can be attributed to its accordion structure, which allows for greater flexibility and adaptation to deviation errors.

Figure 11 highlights the performance of cups (a) and (b) when handling curved objects. Solutions (c) and (d) were excluded due to their inability to form a proper seal on curved surfaces. Solution (c) has cups that are too large, while solution (d) has fixed cups in a grid configuration, both of which fail to adapt to curved shapes effectively. Our adaptive cups (a) again show performance closely matching solution (b). However, solution (b) retains a distinct advantage due to its use of silicone materials, which are softer and more adaptable to positional errors than TPU used to manufacture adaptive cups. Our findings suggest that our adaptive cups, despite being slightly outperformed by solution (b) in certain aspects, still offer grasping robustness against detection error. This makes it suitable for a wide range of applications where precision and adaptability are important. From a broader perspective, these results contribute to the ongoing efforts to enhance the reliability of automated grasping systems. The better performance of solution (b) due to its structural and material advantages suggests potential areas for improvement in our design, such as incorporating more flexible materials.

Figure 12 illustrates the success rates of various grasping cups in combination with our detection pipeline for both flat and curved objects. For flat objects, the data reveal that the highest success rate was achieved with the commercial suction cups (b). This performance can once again be attributed to the accordion structure and material composition of these suction cups. However, our suction cups (a) demonstrated a success rate that was closely comparable to that of the commercial suction cups. This suggests that our design is effective, although there may still be room for optimization to match the performance of cups (b). In contrast, cups (c) had problems with heavier objects. The softness and larger size of its suction cups sometimes failed to provide sufficient holding force, leading to reduced success rates. Cups (d) had the lowest success rate among the tested cups. The primary issue was the small seal size and diameter of suction cups, which often resulted in the grasped objects falling off. This observation underscores the critical importance of appropriate suction cup size and stiffness in ensuring a reliable grip on flat objects. In the case of curved objects, solutions (c) and (d) were excluded from the analysis due to their inherent inability to grasp such shapes effectively. The performance of our suction cups (a) was reasonably good when compared to the commercially available suction cups (b). Although the commercial solution (b) slightly outperformed our suction cups, the difference was not substantial, indicating that our design is capable of handling curved objects. Overall, these results highlight the importance of tailoring suction cup properties—such as size, stiffness, and material composition—to the specific requirements of the objects being grasped. Our solution shows promise in both flat and curved object scenarios, and further refinements could enhance its efficacy to potentially surpass commercially available options.

In a study by Mahler et al. [16], a method is presented that demonstrates superior performance in terms of grasping success rate across nearly all reported statistics (98% success rate when dealing with basic object shapes). However, it is important to note that the scenarios and experimental conditions between their work and ours differ significantly. As such, a direct comparison of performance metrics can be misleading since the variations in experimental setup, object diversity, and grasping conditions may have a considerable impact on the outcomes.

Figure 13 presents two graphs illustrating the data captured during the experimental procedures. The top graph depicts the robot’s velocities across different axes, providing an overview of the robot’s movements throughout the experiment. This visualization helps in understanding the sequence and nature of the robot’s actions. The bottom graph displays the acceleration data collected from the grasped object, offering insights into the dynamic interactions between the robot and the object. In phase (c), the robot is approaching the object in a linear motion, which is evidenced by the acceleration showing a consistent 1 g in the −Y direction, pointing toward the ground. The relatively flat nature of the acceleration graph in this phase indicates that the object remains stationary, unaffected by any external forces other than gravity. Phase (d) marks the grasping maneuver, characterized by pronounced spikes on the acceleration graph. These spikes signify the initiation of contact and the forces exerted by the robot to secure the object, causing sudden changes in acceleration. During phases (e), (f), (g), and (h), the object is in motion. The oscillations and spikes observed in the acceleration graph during these phases reflect the shear forces acting on the object. Notably, phases (f) and (g) involve rotational movements. This rotational motion is evident from the shift in the major acceleration component, which transitions from the −Y direction to the +X direction, indicating a change in the direction of the gravitational acceleration experienced by accelerometer. At the boundary of phases (h) and (i), another significant spike is observed, signifying the moment when the object is dropped onto its designated spot. This abrupt change in acceleration corresponds to the impact forces as the object comes to rest.

In this study, we introduced the grasp shear force resilience (gsfr) metric to evaluate various grasping solutions. Our findings, shown in Figure 14, Figure 15, Figure 16 and Figure 17, provide a comparative analysis of the gsfr metric across different object types, weights, and experimental phases. A higher gsfr value indicates better resilience to shear forces during manipulation. Figure 14 and Figure 15 show gsfr metrics for flat objects weighing 1 kg and 0.5 kg, respectively. The results show that lighter objects yield higher gsfr values, reflecting better resilience to shear forces, as lighter objects exert less force on the grasping mechanism. Notably, suction cups (d) performed best against shear forces, likely due to their rigid structure and arrangement. Our suction cups (a) performed better than (b) and (d) in movements along the Z-axis and rotational axes for the 1 kg object, with statistically significant *p*-values. For the 0.5 kg object, our suction cups were better only in the rotational scenario. Figure 16 and Figure 17 display gsfr metrics for curved objects weighing 1 kg and 0.5 kg. Solutions (c) and (d) were excluded as they could not grasp curved objects. Lighter curved objects also yielded higher gsfr values. Our grasping solution outperformed solution (b) significantly in the 1 kg scenario, demonstrating better shear force resilience. While the results for the 0.5 kg scenario are more ambiguous, our solution still maintains a slight edge over (b).

The introduction of the gsfr metric allows additional evaluation of grasping solutions. Prior research in this field has primarily focused on in-depth analyses of experimental grasping solutions (Correll et al. [17]), novel methods for grasping (Huang et al. [19]), grasp control (Costanzo et al. [28]), and overall system architecture and challenges (Bormann et al. [18]). However, these studies have not implemented a standardized measure to quantify grasp performance. Our gsfr metric addresses this gap by providing a quantitative means to assess the impact of shear forces on different grasping solutions. This metric allows comparative evaluation of various grasping technologies and aids in identifying specific areas for improvement in grasp design and control.

The results show that rigid cups (c) and (d) are not effective for grasping curved objects. In contrast, cups (b), with their accordion structure and silicone material, and our cups (a) with deformation chamber for changing the curvature of the seal, are adaptable and suitable for curved objects. Rigid cups provide a more stable grasp, and our adaptive cups (a) combine both properties. Our experimental results demonstrate that adaptive suction cups outperform commercially available ones in specific scenarios, but improvements in material composition (using softer material such as silicone) could enhance their performance further. Despite these areas for improvement, our suction cups offer the best adaptability and performance for our use case, making them a preferable choice overall.

## 5. Conclusions

The aim of this work is to advance the field of robotic in-store picking and stocking through the proposal of a comprehensive system architecture, the development of a novel adaptive grasping solution, and the introduction of a new metric, grasp shear force resilience, for evaluating grasping solutions. The importance of our proposed framework lies in its modularity, which is essential for developing versatile robotic systems in retail environments. Our novel adaptive suction cups demonstrate advantages over commercially available alternatives, particularly in scenarios characterized by robustness against detection errors. The solution not only performs comparably in such conditions but, in some cases, even surpasses existing commercial solutions. This adaptability and performance show the potential of our approach to enhance robotic manipulation tasks in retail settings. The introduction of the grasp shear force resilience metric extends the analysis of grasping and manipulation in retail environments. This metric provides a meaningful way to evaluate and compare different grasping solutions, particularly when dealing with delicate items. It allows for a more nuanced understanding of the strengths and limitations of various grasping techniques.

Looking ahead, our future work will focus on further refining the technology underlying our adaptive grasping solution. We plan to explore a hybrid approach combining FDM printing with silicone to enhance the performance and durability of the cups as well as produce a version made entirely of silicone. Additionally, we aim to improve the adaptability of our solution by potentially integrating additional vacuum-driven degrees of freedom, which would potentially enable the grasping of a bigger variety of objects. Another critical area of future research is the enhancement of knowledge representation and a reasoning component, which will be vital for enabling more sophisticated and intelligent robotic behaviors.

In addition, we will explore the integration of large language models (LLMs) to enhance social human–robot interaction in retail environments, which allows for advanced capabilities in customer assistance, product recommendations, and task requests. Another avenue for future work involves leveraging LLMs for task segmentation, enabling more intelligent and adaptive task planning in complex retail scenarios. Finally, we will conduct additional tests to evaluate the system as a whole, which ensures that all components work seamlessly together to deliver optimal performance in real-world retail environments as well as in various scenarios such as restocking and order picking in structured and unstructured environments.

Our work lays a strong foundation for the development of advanced robotic systems capable of efficient and reliable in-store picking and stocking. The proposed system architecture, adaptive grasping solution, and novel metric collectively contribute to this goal, highlighting the importance of modularity and evaluation in advancing robotic technology.

## Figures and Tables

**Table 1:** *p*-values for pairs of our novel suction cups and commercially available suction cups. Bold values represent pairs where data are statistically significant. Italic values represent pairs where data are borderline statistically significant. Cells marked with “n. a.” represent pairs that were not tested due to tehnical limitaions.

Movement	Suction Cup Type	0.5 kg Flat	1 kg Flat	0.5 kg Curved	1 kg Curved
Linear X	***b***	1.08 × $10^{-1}$	3.01 × $10^{-1}$	1.56 × $10^{-1}$	**2.26 × 10^−5^**
	***c***	1.51 × $10^{-1}$	2.20 × $10^{-1}$	n. a.	n. a.
	***d***	**5.02 × 10^−5^**	**1.01 × 10^−4^**	n. a.	n. a.
Linear Z	***b***	1.05 × $10^{-1}$	**6.01 × 10^−3^**	*3.57 × 10^−2^*	**4.51 × 10^−3^**
	***c***	*1.10 × 10^−2^*	**6.11 × 10^−3^**	n. a.	n. a.
	***d***	**5.32 × 10^−4^**	**9.42 × 10^−5^**	n. a.	n. a.
Rotational	***b***	*2.49 × 10^−2^*	**1.01 × 10^−6^**	*5.58 × 10^−2^*	**1.02 × 10^−6^**
	***c***	**1.82 × 10^−3^**	**2.21 × 10^−5^**	n. a.	n. a.
	***d***	**1.45 × 10^−5^**	**1.22 × 10^−6^**	n. a.	n. a.

## Data Availability

Data are contained within the article.

## Abbreviations

The following abbreviations are used in this manuscript:

LIDAR	Light Detection And Ranging
PC	Point cloud
TCP	Tool center point
EM	Expectation maximization
RTDE	Real-time data exchange
FDM	Fused deposition modeling
TPU	Thermoplastic polyurethane
PLA	Polylactic acid
gsfr	grasp shear force resilience

## References

[1] Spahn M., Pezzato C., Salmi C., Dekker R., Wang C., Pek C., Kober J., Alonso-Mora J., Hernandez Corbato C., Wisse M. Demonstrating Adaptive Mobile Manipulation in Retail Environments. Proceedings of the Robotics: Science and System XX.

[2] Tonioni A., Serra E., Di Stefano L. A deep learning pipeline for product recognition on store shelves. Proceedings of the 2018 IEEE International Conference on Image Processing, Applications and Systems (IPAS).

[3] Lin T.Y., Goyal P., Girshick R., He K., Dollar P. Focal Loss for Dense Object Detection. Proceedings of the IEEE International Conference on Computer Vision (ICCV) 2017.

[4] Goldman E., Herzig R., Eisenschtat A., Goldberger J., Hassner T. Precise Detection in Densely Packed Scenes. Proceedings of the IEEE/CVF Conference on Computer Vision and Pattern Recognition (CVPR).

[5] Zhang H., Wang Y., Dayoub F., Sunderhauf N. VarifocalNet: An IoU-Aware Dense Object Detector. Proceedings of the IEEE/CVF Conference on Computer Vision and Pattern Recognition (CVPR).

[6] Dimitropoulos N., Papalexis P., Michalos G., Makris S., Wagner A., Alexopoulos K., Makris S. (2024). Advancing Human-Robot Interaction Using AI—A Large Language Model (LLM) Approach. Proceedings of the Advances in Artificial Intelligence in Manufacturing.

[7] Banur O., Patle B., Pawar S. (2024). Integration of robotics and automation in supply chain: A comprehensive review. Robot. Syst. Appl..

[8] Kuhn H., Sternbeck M.G. (2013). Integrative retail logistics: An exploratory study. Oper. Manag. Res..

[9] Miller A., Knoop S., Christensen H., Allen P. Automatic grasp planning using shape primitives. Proceedings of the 2003 IEEE International Conference on Robotics and Automation (Cat. No.03CH37422).

[10] Saxena A., Driemeyer J., Kearns J., Ng A., Schölkopf B., Platt J., Hoffman T. (2006). Robotic Grasping of Novel Objects. Proceedings of the Advances in Neural Information Processing Systems.

[11] Saxena A., Driemeyer J., Kearns J., Osondu C., Ng A.Y. (2008). Learning to Grasp Novel Objects Using Vision. Experimental Robotics: The 10th International Symposium on Experimental Robotics.

[12] Saxena A., Wong L., Quigley M., Ng A.Y., Kaneko M., Nakamura Y. (2011). A Vision-Based System for Grasping Novel Objects in Cluttered Environments. Proceedings of the Robotics Research.

[13] ten Pas A., Gualtieri M., Saenko K., Platt R. (2017). Grasp Pose Detection in Point Clouds. Int. J. Robot. Res..

[14] Liang H., Ma X., Li S., Görner M., Tang S., Fang B., Sun F., Zhang J. PointNetGPD: Detecting Grasp Configurations from Point Sets. Proceedings of the 2019 International Conference on Robotics and Automation (ICRA).

[15] Sundermeyer M., Mousavian A., Triebel R., Fox D. Contact-GraspNet: Efficient 6-DoF Grasp Generation in Cluttered Scenes. Proceedings of the 2021 IEEE International Conference on Robotics and Automation (ICRA).

[16] Mahler J., Matl M., Liu X., Li A., Gealy D., Goldberg K. Dex-Net 3.0: Computing Robust Vacuum Suction Grasp Targets in Point Clouds Using a New Analytic Model and Deep Learning. Proceedings of the 2018 IEEE International Conference on Robotics and Automation (ICRA).

[17] Correll N., Bekris K.E., Berenson D., Brock O., Causo A., Hauser K., Okada K., Rodriguez A., Romano J.M., Wurman P.R. (2018). Analysis and Observations From the First Amazon Picking Challenge. IEEE Trans. Autom. Sci. Eng..

[18] Bormann R., de Brito B.F., Lindermayr J., Omainska M., Patel M., Tzovaras D., Giakoumis D., Vincze M., Argyros A. (2019). Towards Automated Order Picking Robots for Warehouses and Retail. Proceedings of the Computer Vision Systems.

[19] Huang H., Dominguez-Kuhne M., Satish V., Danielczuk M., Sanders K., Ichnowski J., Lee A., Angelova A., Vanhoucke V., Goldberg K. Mechanical Search on Shelves using Lateral Access X-ray. Proceedings of the 2021 IEEE/RSJ International Conference on Intelligent Robots and Systems (IROS).

[20] Miller J.T., Wicks N. (2018). Vacuum-Actuated Bending for Grasping. Robotics.

[21] Xinquan L., Yuzhe W., Zhen X., Ocak S.M. A Vacuum-Powered Soft Mesh Gripper for Compliant and Effective Grasping. Proceedings of the 2023 IEEE International Conference on Soft Robotics (RoboSoft).

[22] Fuchs K., Grundmann T., Fleisch E. Towards Identification of Packaged Products via Computer Vision: Convolutional Neural Networks for Object Detection and Image Classification in Retail Environments. Proceedings of the 9th International Conference on the Internet of Things, IoT ’19.

[23] Guimarães V., Nascimento J., Viana P., Carvalho P. (2023). A Review of Recent Advances and Challenges in Grocery Label Detection and Recognition. Appl. Sci..

[24] Gayathri R., Uma V. (2018). Ontology based knowledge representation technique, domain modeling languages and planners for robotic path planning: A survey. ICT Express.

[25] Paulius D., Sun Y. (2019). A Survey of Knowledge Representation in Service Robotics. Robot. Auton. Syst..

[26] Garcia Ricardez G., Okada S., Koganti N., Yasuda A., Uriguen Eljuri P., Sano T., Yang P.C., El Hafi L., Yamamoto M., Takamatsu J. (2020). Restock and straightening system for retail automation using compliant and mobile manipulation. Adv. Robot..

[27] Işık Ş. (2014). A Comparative Evaluation of Well-known Feature Detectors and Descriptors. Int. J. Appl. Math. Electron. Comput..

[28] Costanzo M., De Maria G., Lettera G., Natale C. (2021). Can Robots Refill a Supermarket Shelf? Motion Planning and Grasp Control. IEEE Robot. Autom. Mag..

